# Work‐related factors among people with diabetes and the risk of cardiovascular diseases: A systematic review

**DOI:** 10.1002/1348-9585.12278

**Published:** 2021-10-02

**Authors:** KM Saif‐Ur‐Rahman, Razib Mamun, Yuanying Li, Masaaki Matsunaga, Atsuhiko Ota, Hiroshi Yatsuya

**Affiliations:** ^1^ Department of Public Health and Health Systems Graduate School of Medicine Nagoya University Nagoya Japan; ^2^ Health Systems and Population Studies Division ICDDRB Dhaka Bangladesh; ^3^ Department of Public Health Fujita Health University School of Medicine Toyoake Aichi Japan

**Keywords:** cardiovascular diseases, diabetes, occupational health, systematic review, work‐related factors

## Abstract

**Background:**

Diabetes is a major risk factor for cardiovascular diseases (CVD). This systematic review aims to explore the work‐related factors among people with diabetes in developing CVD.

**Methods:**

Four electronic databases were searched on 1 February 2021 using a comprehensive search strategy without any time restriction. Two independent researchers screened the articles and extracted data. The risk of bias was assessed independently using the risk of bias assessment tool for non‐randomized studies (RoBANS). A narrative synthesis was conducted considering the heterogeneity of the included articles.

**Results:**

A total of five articles incorporating 4 409 810 participants from three geographic regions were included that highlights the research gap. As per the included studies, Occupational drivers with diabetes were at a higher risk of CVD in comparison to the nondrivers, workers with diabetes having a long working hour were at a higher risk of CVD mortality, workers with a lower occupational status were at a higher risk of 10‐years stroke risk, and occupational physical activity and occupational commuting lowered the risk of CVD deaths.

**Conclusions:**

This systematic review summarized the available evidence on work‐related factors influencing the risk of CVD in people with diabetes. The findings should be interpreted cautiously pondering the limited evidence and imprecision. We identified only five articles related to the topic, and there were no studies from Japan. The scarcity of studies on work‐related factors on the prognosis of diabetic patients implies the need for more research in this field. We recommend further exploration of the topic designing primary studies.

## 1 INTRODUCTION

The safety of workers with comorbidities is an important issue in occupational health. The number of workers with diabetes is increasing due to the aging of the global society.^1^, ^2^ A number of occupational and work‐related factors have been reported to increase the risk of developing cardiovascular disease (CVD).^3^, ^4^, ^5^, ^6^, ^7^ Diabetes is known as one of the major risk factors of CVDs,^8^ which is the leading cause of death globally and explains a significant proportion of the global burden of disease.^9^ However, little is known specific occupational factors in relation to CVD risk in workers with diabetes.

Prioritizing the safety of workers by tailoring to individual risks of developing life‐threatening diseases while making the best use of all the workforces regardless of comorbidities would be important to create a future norm of occupational health practices and to achieve the sustainable development goals (SDG) 3 on good health and well‐being.^10^

Therefore, it was considered important to collect currently available evidence and suggest the need for future study if necessary, regarding work‐related factors among workers with diabetes in developing CVDs. In the present report, we conducted a systematic review with an aim to summarize all the relevant scientific articles demonstrating the work‐related factors contributing to the development of CVDs among workers with diabetes.

## 2 MATERIAL AND METHODS

The systematic review followed the standard guidelines of preferred reporting items for systematic review and meta‐analysis (PRISMA).^11^ The protocol of the systematic review has been registered with PROSPERO (International prospective register of systematic reviews). The systematic review registration number is CRD42021223807.

Three electronic databases (Medline through PubMed, Web of Science, and Cochrane library) were searched using a comprehensive search strategy on February 1, 2021, and updated on August 15, 2021. In addition, we searched the Japanese database “ICHUSHI” for relevant Japanese articles. The search strategy was developed in consultation with a systematic review expert, occupational health expert, and metabolic disorder expert. Key search terms were developed considering the population, intervention, and outcome of the review question. All possible relevant and synonymous terms were considered. All the terms were combined using the Boolean operators. Besides, an extensive citation tracking of the included articles was conducted. The comprehensive search strategy for different databases has been demonstrated in Table 1. The search strategy used in ICHUSHI has been provided in Table S1.

**TABLE 1:** Comprehensive search strategy for different databases

	Search terms
PubMed search	
1	“cardiovascular diseases” [Title/Abstract] OR “cardiovascular diseases” [MeSH] OR CVD [Title/Abstract] OR CVD [MeSH] OR “coronary artery disease” [Title/Abstract] OR “coronary heart disease” [Title/Abstract] OR stroke [Title/Abstract] OR “cerebral Infarction” [Title/Abstract] OR “intracerebral hemorrhage” [Title/Abstract] OR "myocardial infarction" [Title/Abstract] OR "brain infarction” [Title/Abstract]
2	work‐related [Title/Abstract] OR work‐related [MeSH] OR “occupational” OR “job‐related” OR "cardiovascular risk factors" OR workload [Title/Abstract] OR "work support" [Title/Abstract] OR "work demands" [Title/Abstract] OR "work pressure" [Title/Abstract] OR "shift work" [Title/Abstract] OR "long working hours" [Title/Abstract] OR stress [Title/Abstract] OR "physical activity" [Title/Abstract] OR "effort reward imbalance" [Title/Abstract] OR "professional role" [Title/Abstract] OR "Occupational Stress" [Title/Abstract] OR "Work Schedule" [Title/Abstract] OR "Overtime work" [Title/Abstract]
3	“Type 2 diabetes mellitus” [Title] OR T2DM [Title] OR "Diabetes mellitus" [Title] OR Diabetes [Title] OR DM [Title]
4	1 AND 2 AND 3
Web of Science search	
1	“cardiovascular diseases” OR CVD OR “coronary artery disease” OR “coronary heart disease” OR stroke OR “cerebral Infarction” OR “intracerebral hemorrhage” OR "myocardial infarction" OR "brain infarction” Filter: None, Topic
2	work‐related OR “occupational” OR “job‐related” OR "cardiovascular risk factors" OR workload OR "work support" OR "work demands" OR "work pressure" OR "shift work" OR "long working hours" OR stress OR "physical activity" OR “effort reward imbalance” OR “professional role” OR "Occupational Stress" OR "Work Schedule" OR "Overtime work" Filter: None, Topic
3	“Type 2 diabetes mellitus” OR T2DM OR "Diabetes mellitus" OR Diabetes OR DM Filter: Title
4	1 AND 2 AND 3
Cochrane library search	
1	“cardiovascular diseases” OR CVD OR “coronary artery disease” OR “coronary heart disease” OR stroke OR “cerebral Infarction” OR “intracerebral hemorrhage” OR "myocardial infarction" Filter: tiab, keyword
2	work‐related OR “occupational” OR “job‐related” OR "cardiovascular risk factors" OR workload OR "work support" OR "work demands" OR "work pressure" OR "shift work" OR "long working hours" OR stress OR "physical activity" OR “effort reward imbalance” OR “professional role” OR "Occupational Stress" OR "Work Schedule" OR "Overtime work" Filter: tiab, keyword
3	“Type 2 diabetes mellitus” OR T2DM OR "Diabetes mellitus" OR Diabetes OR DM Filter: Record title
4	1 AND 2 AND 3

Prespecified inclusion and exclusion criteria were used to include the retrieved articles. The inclusion criteria were as follows: (1) the study population was people with diabetes, (2) any article demonstrating the work‐related factors influencing the development of CVD among workers with diabetes were included, (3) there was no restriction on age, gender, or ethnicity, or geographic region, (4) people of all socioeconomic classes were considered, (5) all study designs including randomized controlled trials, nonrandomized trials, analytical studies, descriptive studies, qualitative studies were considered, (6) all the articles available from the inception of the databases were considered, (7) articles written in any language were included. Work‐related factors included workload, work demand, job control, support, reward, shiftwork, long working hours, exposure to heat, noise, and chemicals.

The exclusion criteria were as follows: (1) study conducted on nondiabetic people, (2) studies not considering the work‐related factors, (3) studies not considering CVD risk of workers with diabetes, (4) letters to the editor, comments, editorial, as they usually do not contain primary results.

At first, retrieved articles were imported to EndNote software (Clarivate). After removing the duplicates, two review authors independently screened the title and abstracts of the articles applying the above‐mentioned inclusion and exclusion criteria. Thereafter, the full texts of the articles included by the previous screening were thoroughly read by each of the two authors. Any conflicting decision was resolved through discussion, and when necessary, the lead reviewer intervened.

Data were extracted by two independent coders. Relevant information on year of publication, country, and geographic region, study design, sample size, study population characteristics in terms of age and gender, study period, main outcome related to work‐related factors among diabetic patients having CVDs, etc. were extracted. Two independent coders discussed and matched the extracted information.

The quality of the included articles was assessed by two independent review authors using the Joanna Briggs Institute (JBI) quality assessment tool.^12^ The JBI offers a set of critical appraisal checklists for different study designs. The main focus of the appraisal checklist is on methodological rigor, avoidance of bias including selection bias, and information bias, appropriate use of statistics, and appropriate reporting, etc. The total number of quality assessment items was 9 for a cross‐sectional study, 8 for a comparative cross‐sectional study, and 11 for a cohort study. We assigned 1 point if the response for a specific item was “Yes,” or “Not applicable.” No pints were allocated if the responses were “No,” or “Unclear.” The total quality score was calculated by summation of the score for each item provided in the quality assessment checklist. Additionally, the risk of bias of the included studies was assessed using the “risk of bias assessment tool for nonrandomized studies” (RoBANS).^13^ The RoBANS tool focuses on six major domains to address selection bias, performance bias, detection bias, attrition bias, and reporting bias.

A narrative synthesis was conducted summarizing the results of included articles. A quantitative synthesis or meta‐analysis was not feasible due to the heterogeneity of the included articles. The certainty of the evidence was not assessed as there was no quantitative pooling of results. Due to the limited number of included articles, publication bias was not assessed.^14^

## 3 RESULTS

**FIGURE 1:**
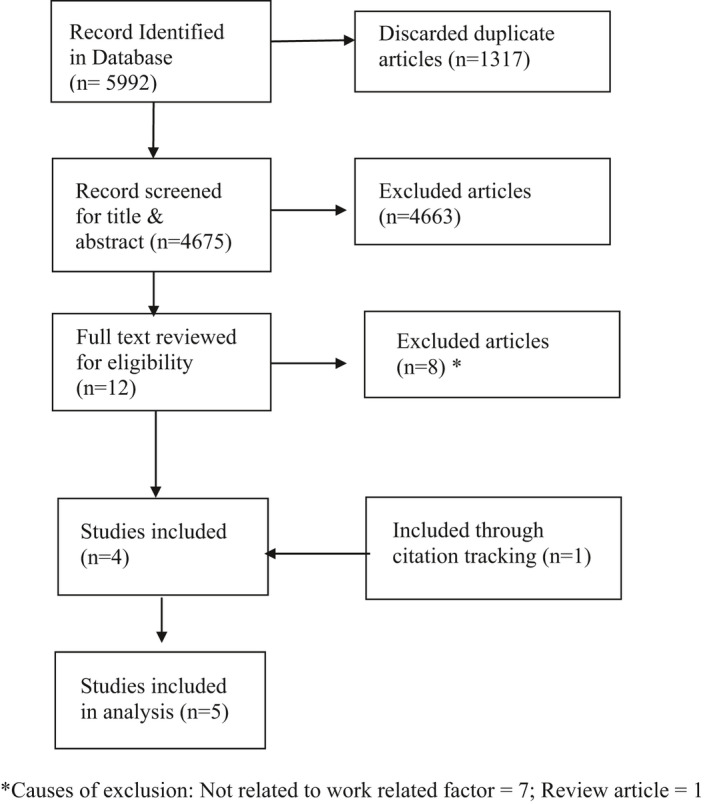
Systematic review flow diagram

The comprehensive search of the databases retrieved 5992 articles. After removing 1317 duplicates, the remaining 4675 articles were screened for eligibility. At the title and abstract screening phase, 4663 articles were excluded as they did not meet the inclusion criteria. Among the remaining 12 articles, 8 were excluded during the full‐text screening phase. The causes of exclusion were, not related to work‐related factors (*n* = 7) and traditional review articles (*n* = 1). One article was included through citation tracking. In total, 5 articles were included for the final analysis. The selection process with the numbers and reasons for exclusion has been demonstrated in Figure 1.

The included articles were published between 2004 and 2021. All five studies were conducted in high‐income countries. The geographic region covered the region of the Americas (United States of America),^15^ European region (Finland, Sweden),^3^, ^16^ and Western Pacific region (Singapore, Republic of Korea).^17^, ^18^ The study design of the included articles was cross‐sectional, comparative cross‐sectional, and cohort study. However, the comparative cross‐sectional study was published as a brief report.^17^ The study period ranged from 1983 to 2016. All the studies incorporated both men and women. The age range of the participants was 25 to 80 years. In total, data of 4 409 810 participants were analyzed in the 5 included studies. We intended to include articles written in English and Japanese language. However, we did not get any article other than in English. Characteristics of included articles have been described in Table 2.

**TABLE 2:** Characteristics of included articles, summary of the main findings, and their quality assessment

Author, publication year	Region; country	Study design; sample size	Study population (age, gender)	Study period	Main findings related to work‐related factors among diabetic patients having cardiovascular diseases	Quality assessment score
Hu et al, 2004 ^3^	Finland	Cohort study; 3316	Age range 25–74 years; both men and women	Mean follow‐up of 18.4 years, followed up until the end of 2001	‐ Participants reporting two or three types of moderate to high physical activity had a 45%–48% decreased risk for total mortality and a 44%–48% decreased risk for CVD mortality in comparison to the participants with low level of occupational physical activity, commuting, and leisure‐time physical activity	11/11
Young et al, 2019 ^15^	USA	Cross‐sectional study; 48	Mean age 61.79 years, age range 40–80 years; both men and women	2013–2014	‐ In the lower occupational status score subgroup, the associations between total PAID scores and estimated 10‐year stroke risks were statistically significant (*p* <0.05). ‐ No such statistically significant associations were observed in the higher occupational status score subgroup.	5/9
Carlsson et al, 2021^16^	Sweden	Cohort study; 4,398,117	Age range 35–65; both men and women	2001–2015	‐ Age‐standardized CVD mortality was 384 and 179, respectively, in men and women workers with diabetes. ‐ Absolute 10‐year CVD mortality risk was highest among manufacturing laborers both in men (HR 8.7, 95% CI: 6.4–11.3) and women (HR 3.3, 95% CI: 1.7–5.3).	11/11
Quah et al, 2012^17^	Singapore	Comparative cross‐sectional study; 1026	Mean age of drivers 52.9 years; Mean age of nondrivers 52.8 years; both men and women	2004–2010	‐ Overall risk of a CVD event in occupational drivers compared with nondrivers was increased (OR 1.92, 95% CI: 1.46–2.51)	8/8
Lee et al, 2021^18^	Republic of Korea	Cohort study; 7303	Age range 21–60 years; both men and women	2009–2016	‐ An increased risk of CVD among workers with diabetes who had long working hours (HR 1.47, 95% CI: 1.02–2.11) ‐ The risk significantly attenuates after adjusting for age, educational level, household income level, type of work, and occupational classification (HR 1.07, 95% CI: 0.74–1.54)	11/11

Abbreviation: CVD, cardiovascular disease.

PAID score: A questionnaire used to measure the participants’ distress levels specifically relating to diabetes management.

The quality of the included studies was assessed using the JBI critical appraisal checklist for prevalence studies, analytic cross‐sectional studies, and cohort studies. The cross‐sectional study^15^ used an appropriate sampling frame, described the study settings in detail, used appropriate and reliable statistical methods. However, the sampling technique was not appropriate, the sample size was not adequate, the analysis did not cover a sufficient proportion of the identified sample, and the response rate was not adequate. Though the comparative cross‐sectional study was a brief report,^17^ it described the study participants, measurement of exposure, confounders sufficiently, and used appropriate statistical techniques. The cohort studies^3^, ^16^, ^18^ were also rated as high quality due to well‐reported characteristics and methodological rigor.

As per the RoBANS, the selection bias is assessed considering the selection of participants and confounding variables. The selection of participants was graded as high risk of bias in the cross‐sectional studies,^15^, ^17^ whereas graded as low risk of bias in the cohort studies.^3^, ^16^, ^18^ All five studies measured the confounders and exposures appropriately and were ranked as low risk of bias. None of the studies mentioned the blinding of outcome assessment and therefore, ranked as unclear risk of bias. There was no incomplete outcome data and selective reporting in any of the studies. Thus, the studies were ranked as low risk of bias for attrition bias and reporting bias. Table 3 demonstrates the risk of bias of included studies as per the individual domains.

**TABLE 3:** Risk of bias assessment of included studies

Author, publication year	Domains of risk of bias assessment tool for nonrandomized studies (RoBANS)					
	Selection of participants (selection bias)	Confounding variables (selection bias)	Measurement of exposure (performance bias)	Blinding of outcome assessment (detection bias)	Incomplete outcome data (attrition bias)	Selective outcome reporting (reporting bias)
Hu et al, 2004^3^	Low	Low	Low	Unclear	Low	Low
Young et al, 2019^15^	High	Low	Low	Unclear	Low	Low
Carlsson et al, 2021^16^	Low	Low	Low	Unclear	Low	Low
Quah et al, 2012^17^	High	Low	Low	Unclear	Low	Low
Lee et al, 2021^18^	Low	Low	Low	Unclear	Low	Low

Yong et al explored the association between diabetic distress and cardiovascular complications in California.^15^ The authors used the Nam‐Powers‐Boyd scale to define the occupational status score. They also utilized the PAID (Problem Areas in Diabetes) questionnaire to measure the diabetes‐related distress of the participants. In subgroup analysis, stratified for socioeconomic status, authors reported that there is a significant association between 10‐years stroke risk (coefficient 0.635, 95% CI 0.0088–1.26) and PAID score among the participants belonging to the lower occupational status. The authors reported that higher diabetic distress was significantly associated with predicted 10‐year stroke risk (both fatal and nonfatal) in this group. However, among the higher occupational status group, there was no such significant association.

Qua et al identified occupational driving as a potential risk of CVD among people with diabetes in Singapore.^17^ The comparative study was conducted among the cohort of workers with diabetes receiving care from a general hospital. Multivariable regression analysis adjusted for age, sex, ethnicity, diseases (diabetes) duration, and CVD risk factors, revealed that the occupational drivers are at higher risk of developing CVD events (OR 1.92, 95% CI: 1.46–2.51) in comparison to the nondrivers. The authors discussed some factors such as long working hours, lower physical activity, shift work, exposure to noise, exposure to chemicals including carbon monoxide and nitrous oxide, etc. as the potential explanation of this association among drivers.

Hu et al analyzed the data from the Finnish cohort study to explore the association of occupational activity, commuting activity, and leisure‐time activity with cardiovascular mortality among diabetic patients.^3^ Multivariable analysis adjusted for age, sex, study year, BMI, systolic blood pressure, serum cholesterol level, and smoking status showed the significant hazard ratio with moderate (HR 0.84, 95% CI 0.70–1.01) and active (HR 0.59, 95% CI 0.50–0.69) occupational physical activity. Similarly, there was a significant association with participants who spent more than 30 min on walking or cycling (HR 0.74, 95% CI 0.61–0.90) as a commuting method. Moderate (HR 0.85, 95% CI 0.74–0.98) and high (HR 0.70, 95% CI 0.51–0.96) levels of leisure‐time physical activity were also significantly associated with CVD mortality among workers with diabetes.

Carlsson et al included the employees in Sweden to explore the occupation that poses a higher risk of CVD among workers with diabetes.^16^ Among male workers with diabetes, the manufacturing laborers were at the highest 10‐year CVD mortality risk (HR 8.7, 95% CI: 6.4–11.3), 10‐year IHD risk (HR 24.7, 95% CI: 20.4–29.3), and 10‐year stroke risk (HR 9.6, 95% CI: 7.0–12.7) compared to other workers. The manufacturing laborers were at the highest 10‐year CVD mortality risk (HR 3.3, 95% CI: 1.7–5.3), 10‐year IHD risk (HR 11.5, 95% CI: 7.7–16.2), and 10‐year stroke risk (HR 4.6, 95% CI: 2.5–7.3) compared to other professions among female workers with diabetes as well. College and university teachers were at the lowest risk among the male, and health professionals were at the lowest risk among female workers with diabetes.

Lee et al examined the effect of long working hours on developing risk of CVD^18^ among Korean workers. In a subgroup analysis, the authors have demonstrated the workers with diabetes who had a long working hour were at a higher risk of CVD mortality (HR 1.47, 95% CI: 1.02–2.11). However, the association was attenuated when they adjusted for age, educational level, household income level, type of work (paid and self‐employed), and occupational classification (as per international standard classification of occupation). The study did not report HR adjusted only for nonoccupational factors.

## 4 DISCUSSION

This systematic review identified five observational studies conducted in three different geographic regions exploring the work‐related factors associated with CVD risk among diabetic people. The overall quality of the included articles was good except for one cross‐sectional study,^15^ which failed to express methodological rigor when assessed critically. Work‐related factors such as lower occupational status,^15^, ^16^ occupational driving,^17^ manufacturing work,^16^ long working hours^18^ were found to be associated with CVD among diabetic patients. Occupational physical activity and occupational commuting decreased the risk of CVD mortality.^3^ The quality of the included cross‐sectional study was downgraded due to inadequate sample size, improper sampling technique, and inadequate response rate. The comparative cross‐sectional study and the cohort studies reported all methodological concerns precisely and provided an impression of good quality. The cross‐sectional study and the comparative cross‐sectional study were ranked as high risk of selection bias due to an inadequate selection of participants. Detection bias was unclear in all three studies. Performance bias, attrition bias, and reporting bias were considered low in all three studies. A meta‐analysis of different associations was not possible to conduct due to the heterogeneity of the exposure.

A comprehensive search was used to get all available articles related to the objective of the systematic review. The review authors did not get any systematic review exploring the work‐related factors among diabetic patients related to the risk of CVD. In contrast, work‐related factors as a risk of CVD is relatively well‐researched issue among workers in general.^19^, ^20^, ^21^, ^22^, ^23^ Researchers have identified both environmental and psychosocial CVD risk factors among workers.^19^ The risk of CVD in diabetes patients is also well researched. A review article identified factors influencing the risk of CVD in diabetic patients.^24^ However, the authors discussed factors such as age, sex, family history, presence of metabolic syndrome, duration of diabetes, etc. No factors associated with the occupation were explored or discussed. Another systematic review explored the association of depression with CVD among the diabetic population.^8^ The authors conducted a meta‐analysis of 17 studies that revealed that there is a higher risk of fatal CVD (RR 1.47, 95% CI 1.21–1.77) among depressive diabetic patients.

Our systematic review identified occupational driving as a risk factor of CVD in workers with diabetes.^17^ The long working hours, night shift works, and noise exposure were noted as possible influencing factors. The long working hours were also mentioned as a risk factor of CVD among diabetic workers in another included study.^18^ A Japanese cohort study depicted that the risk of night shift work is associated with higher death due to cardiac diseases (RR = 2.32, 95% CI: 1.37–3.9).^25^ Another study from Japan demonstrated the association of over‐work with CVD. However, that study did not mention the workers with diabetes.^23^ Similarly, a Finnish cohort study showed a higher risk of CVD among shift workers.^26^ The same study highlighted the impact of noise exposure with a higher risk of CVD among workers. Another study conducted a quality effect model meta‐analysis and highlighted that people with higher noise exposure are at a 22% higher risk of having diabetes. However, they did not find any significant association with occupational noise exposure.^27^ There was no description of the association between noise exposure and the risk of CVD among diabetic people in our included studies.

Our systematic review identified the effect of occupational physical activity and occupational commuting among workers with diabetes in CVD mortality in a cohort study. We did not get any such findings from other literature. However, a meta‐analysis of 17 studies reflected that the 1‐unit increment of physical activity reduced the risk of CVD by 7.9%.^28^ Another study conducted in California demonstrated that walking more than an hour reduces the risk of death from non‐CHD (non‐coronary heart disease) CVD among diabetic people.^29^ Besides, the same group of authors described the association of these factors in CVD mortality as a whole^7^ and among hypertensive people.^4^ We explored that leisure‐time physical activity reduces the risk of CVD mortality, as stated by Hu et al in their article. Similarly, the Fukuoka diabetic registry in Japan reported that leisure‐time physical activity is associated with good glycemic control and lower risk of CVD.^30^

One of the included studies in this review depicted the association between lower occupational status among diabetic people with stroke mortality.^15^ Similarly, another included study concluded that the manufacturing laborers have a higher CVD mortality risk.^16^ An Iranian study reported the highest risk of IHD and stroke among the lower occupational category (clerical support worker).^22^ However, the researchers did not explore the CVD risk among workers with diabetes in that study. The finding of our systematic review would be consistent with a previous study that reported higher diabetic distress was significantly associated with stroke motility.^15^ It was also reported that occupational work stress is linked with metabolic disorders such as hypertension, diabetes, dyslipidemia.^31^

Diabetes is considered one of the major risks of CVDs. Work‐related factors were notified as influencing factors of diabetes and CVD. We hypothesized that work‐related factors might exaggerate the atherosclerotic process or trigger the onset of CVD in diabetic patients. However, we did not get that much evidence in this regard. In addition, the pathological pathway between work‐related factors and CVD in diabetic patients remains largely unknown. This is, in reality, convincing that there is a huge scope of conducting research in this area. Further studies exploring the work‐related factors among diabetic people in developing CVD are certainly required. At the same time, ongoing diabetic cohorts, occupational cohorts, and cardiovascular cohorts might think of incorporating such variables in their ongoing studies to get evidence in this regard. We identified occupational driving as a risk of CVD in workers with diabetes. Experimental studies might be necessary to confirm the finding incorporating interventions to reduce long working hours, shift work, and noise exposure among diabetic drivers. However, such interventions would require surrogate measures of the outcomes both from practical and ethical standpoints. Further studies should have commissioned to explore the impact of occupational physical activity, occupational commuting, and leisure‐time physical activity in reducing the risk of CVD or CVD mortality among workers with diabetes. Future studies should consider occupational status to differentiate the impact of different exposures and interventions between high occupational status and low occupational status groups.

Considering the limited amount of evidence revealed through this systematic review, we are not certain to advocate at the policy level. However, based on the findings of one included study, we may recommend reviewing the work policy of occupational drivers. There should be options to avoid long working hours, shift work, and noise exposure among the workers suffering from diabetes. Our findings also suggest that there should regular health evaluation of workers to reduce any unwanted disease progression.

The methodological robustness is the main strength of this systematic review. We conducted the different stages of the review engaging two independent review authors to minimize any sort of subjectivity. A comprehensive search of three major databases provided us a broader search outcome which minimized the chance of missing any potential relevant article. There was no restriction in search date and language which also ensured a wide range of coverage. However, this systematic review is not devoid of limitations. Only five articles were eligible for inclusion which did not allow us to dig deep into the topic. As a meta‐analysis was not conducted and the certainty of the evidence was not assessed, the findings from this systematic review should be interpreted cautiously to avoid any over grading of evidence. The scarcity of evidence on work‐related factors triggering CVDs among workers with diabetes implies the importance of further research on this topic in Japan and other countries.

## CONFLICT OF INTEREST

The authors declare no conflict of interest.

## AUTHOR CONTRIBUTION

KMSUR: Conceptualization, data curation, formal analysis, methodology, project administration, software, validation, visualization, writing—original draft, writing—review & editing. RM: Data curation, formal analysis, software, validation, writing—review & editing. YL: Writing—review & editing. MM: Data curation, writing—review & editing. AO: Methodology, validation, writing—review & editing. HY: Conceptualization, methodology, project administration, resources, software, supervision, validation, visualization, writing—original draft, writing—review & editing.

## DISCLOSURE

*Ethical approval*: Not applicable. *Informed consent*: Not applicable. *Registry and the Registration No. of the study/Trial*: The protocol of the systematic review has been registered with PROSPERO (International prospective register of systematic reviews). The systematic review registration number is CRD42021223807. *Animal Studies*: Not applicable.

## Supporting information

Table S1

## Data Availability

Availability of template data collection forms; data extracted from included studies; data used for all analyses; analytic code; any other materials used in the review: Available from the corresponding author on request.
