# Supplementary material for: Work‐related factors among people with diabetes and the risk of cardiovascular diseases: A systematic review
Source: J Occup Health. 2021 Oct 2;63(1):e12278. doi: 10.1002/1348-9585.12278 (PMC8487164; doi:10.1002/1348-9585.12278)
Supplement: Supplementary file 1 — Table S1 [file JOH2-63-e12278-s001.docx]

| Search number | **Search terms** |
| --- | --- |
| 1 | ((心臓血管疾患/TH or 心血管疾患/AL or 循環器疾患/AL) or (CVD/AL) or (冠状動脈硬化症/TH or 冠血管疾患/AL) or (冠動脈疾患/TH or 冠動脈心疾患/AL or 冠動脈性心疾患/AL or 虚血性心疾患/AL) or (脳卒中/TH) or (脳梗塞/TH or 大脳梗塞/AL) or (大脳出血/TH or 脳出血/AL or 脳内出血/AL) or (心筋梗塞/TH) or (脳梗塞/TH or 脳梗塞症/AL)) |
| 2 | ((職業関連/AL or 労働関連/AL or 仕事関連/AL) or (労働量/TH or 作業負荷/AL or 作業量/AL or 仕事量/AL) or (作業支援/AL or 労働支援/AL or 仕事支援/AL or 作業サポート/AL or 労働サポート/AL or 仕事サポート/AL or ワークサポート/AL) or (労働要求/AL or 作業要求/AL or 仕事要求/AL) or (労働圧力/AL or 作業圧力/AL or 仕事圧力/AL or 労働負荷/AL or 作業負荷/AL or 仕事負荷/AL) or (交代制勤務/TH or シフトワーク/AL or 交代勤務/AL or 夜勤/AL) or (長時間労働/AL) or (ストレス/TH) or (身体活動/AL or 運動/AL) or (努力－報酬不均衡/AL or 努力報酬不均衡/AL) or (職業的役割/AL or 仕事役割/AL) or (職業ストレス/AL or 職業性ストレス/AL or 仕事ストレス/AL) or (勤務スケジュール/AL or 勤務予定/AL or 労働時間/AL) or (時間外労働/AL or 残業/AL)) |
| 3 | ((糖尿病-2型/TH or ２型糖尿病/AL) or (糖尿病-2型/TH or T2DM/AL) or (糖尿病/TH or DM/AL)) |
| 4 | 1 AND 2 AND 3 |

Supplementary Table 1: Comprehensive search strategy for the ICHUSHI databases
